# Psychological impact of lung cancer screening using a novel antibody blood test followed by imaging: the ECLS randomized controlled trial

**DOI:** 10.1093/pubmed/fdac032

**Published:** 2022-03-14

**Authors:** J Hancox, K Ayling, L Bedford, K Vedhara, J F R Roberston, B Young, R das Nair, F M Sullivan, S Schembri, F S Mair, R Littleford, D Kendrick

**Affiliations:** Centre for Academic Primary Care, Lifespan and Population Health, School of Medicine, University of Nottingham, Applied Health Research Building, University Park, Nottingham, NG7 2RD; Centre for Academic Primary Care, Lifespan and Population Health, School of Medicine, University of Nottingham, Applied Health Research Building, University Park, Nottingham, NG7 2RD; Department of Family Medicine and Primary Care, Li Ka Shing Faculty of Medicine, University of Hong Kong, Hong Kong SAR, China; Centre for Academic Primary Care, Lifespan and Population Health, School of Medicine, University of Nottingham, Applied Health Research Building, University Park, Nottingham, NG7 2RD; Division of Medical Sciences and Graduate Entry Medicine, School of Medicine, University of Nottingham, DE22 3DT Derby, UK; Centre for Academic Primary Care, Lifespan and Population Health, School of Medicine, University of Nottingham, Applied Health Research Building, University Park, Nottingham, NG7 2RD; Division of Psychiatry and Applied Psychology, School of Medicine, University of Nottingham, NG7 2TU Nottingham, UK; School of Medicine, University of St Andrews, KY16 9TF St Andrews, UK; Respiratory Medicine, NHS Tayside, DD2 1UB Dundee UK; Institute of Health & Wellbeing, University of Glasgow, G12 8RZ Glasgow, UK; Centre for Clinical Research, University of Queensland, 4072 Saint Lucia, Australia; Centre for Academic Primary Care, Lifespan and Population Health, School of Medicine, University of Nottingham, Applied Health Research Building, University Park, Nottingham, NG7 2RD

**Keywords:** behavioural medicine, early detection of cancer, lung neoplasms, psychology, screening

## Abstract

**Background:**

The Early CDT^®^-Lung antibody blood test plus serial computed tomography scans for test-positives (TPGs) reduces late-stage lung cancer presentation. This study assessed the psychological outcomes of this approach.

**Methods:**

Randomized controlled trial (*n* = 12 208) comparing psychological outcomes 1–12 months post-recruitment in a subsample (*n* = 1032) of TPG, test-negative (TNG) and control groups (CG).

**Results:**

Compared to TNG, TPG had lower positive affect (difference between means (DBM), 3 months (3m: −1.49 (−2.65, − 0.33)), greater impact of worries (DBM 1m: 0.26 (0.05, 0.47); 3m: 0.28 (0.07, 0.50)), screening distress (DBM 1m: 3.59 (2.28, 4.90); 3m: 2.29 (0.97, 3.61); 6m: 1.94 (0.61, 3.27)), worry about tests (odds ratio (OR) 1m: 5.79 (2.66, 12.63) and more frequent lung cancer worry (OR 1m: 2.52 (1.31, 4.83); 3m: 2.43 (1.26, 4.68); 6m: 2.87 (1.48, 5.60)). Compared to CG, TPG had greater worry about tests (OR 1m: 3.40 (1.69, 6.84)). TNG had lower negative affect (log-transformed DBM 3m: −0.08 (−0.13, −0.02)), higher positive affect (DBM 1m: 1.52 (0.43, 2.61); 3m: 1.43 (0.33, 2.53); 6m: 1.27 (0.17, 2.37)), less impact of worries (DBM 3m: −0.27 (−0.48, −0.07)) and less-frequent lung cancer worry (OR 3m: 0.49 (0.26, 0.92)).

**Conclusions:**

Negative psychological effects in TPG and positive effects in TNG were short-lived and most differences were small.

## Background

Lung cancer is the leading cause of cancer-related deaths worldwide^1^ and accounts for around 35 000 deaths annually in the UK.^2^ Serial computed tomography (CT) screening has been shown to reduce lung cancer mortality by 16%.^3^ CT screening is resource-intensive and results in radiation exposure, a high false positive rate, over-diagnosis, anxiety and distress.^4^^,^^5^ There is currently no national screening programme for lung cancer in the UK, although a targeted Lung Health Check programme, including a risk assessment and CT scan, for those at increased risk of lung cancer is offered in some areas and is currently undergoing evaluation. The limitations of CT screening have fuelled interest in other approaches, including biomarker blood tests to identify those who may benefit from CT screening. The EarlyCDT-Lung^®^ blood test measures antibodies to a panel of lung cancer antigens and has been shown to reduce late-stage presentation of lung cancer by 36%.^6^ This paper reports the psychological impact of using this novel approach, prior to imaging (chest x-ray (CXR) and CT) for those with a positive blood test.

Systematic reviews report CT screening (without use of biomarkers) is associated with increased anxiety and cancer-specific distress for those with true positive results.^7^^,^^8^ The National Lung Screening Trial (NLST) and UK Lung Cancer Screening (UKLS) trial found higher-state anxiety in those with true positive than negative results.^9^^,^^10^ The UKLS also found higher levels of cancer distress 2 weeks post-scan for those with true positive than negative results.^10^

The psychological impact on those with false positive, incidental findings or negative results is less clear. The NLST found no differences between these three groups in state anxiety at 1 or 6 months post-screening.^9^ The Dutch–Belgian Randomised Lung Cancer Screening Trial (NELSON) found short-term increases in state anxiety and cancer-specific distress 2 months post-screening in participants with indeterminate results.^11^

Most studies do not report adverse psychological impacts of lung cancer CT screening beyond 6 months post-screening.^7^^,^^8^ The NELSON trial reported no differences between groups in cancer distress at 1.5 years^12^ and the UKLS trial reported clinically unimportant higher levels of anxiety and depression in the control group (CG) than the screened group up to 2 years post-screening.^10^ By contrast, the Danish Lung Cancer Screening Trial (DLCST) reported greater negative impact of annual CT screening (e.g. higher levels of anxiety and feelings of dejection) in the CG compared to the screened group at 1, 2 and 4 years,^13^^,^^14^ possibly due to reassurance from the negative results in those screened. Given that most people screened receive negative results, this may indicate psychological benefits of screening. Previous lung cancer screening research has only measured negative psychological outcomes; hence, it is unclear whether screening results in positive psychological outcomes (e.g. positive affect). This paper compares the psychological impact of EarlyCDT-Lung^®^ screening, followed by imaging for those with positive blood tests, between test-positive, test-negative and CG participants in the short (≤6 months) and longer term (>6 months).

## Method

### Trial design

Parallel group randomized controlled trial, with participants allocated in a 1:1 ratio to intervention (blood test, followed by imaging for those with a positive blood test) and control (no blood test) groups.

### Participants

Participants were recruited in the Greater Glasgow and Clyde, Tayside and Lanarkshire Scottish National Health Service regions, predominantly from general practices and also by self-referral. Inclusion criteria were: aged 50–75 years, current or ex-smokers, smoking history of ≥20 pack years or first-degree relative with lung cancer, Eastern Cooperative Oncology Group performance status of 0–2 and able to give informed consent. Exclusion criteria were: history of any cancer (excluding non-melanomatous skin cancer, cervical cancer *in situ*), symptoms suggestive of lung cancer within the last 6 months, terminal disease, >3 months of continuous use of cyclophosphamide or if their General Practitioner felt trial invitation would cause undue distress.^15^

A subsample of trial participants from Greater Glasgow and Clyde and Tayside were invited, by mail, to complete questionnaires assessing psychological responses to lung cancer screening from January 2014 to May 2015. All positive test participants were invited. Random samples of 21 individuals per week from each of the negative test and CGs were invited (all were invited, where <21 per week). Participants completed questionnaires at 1, 3, 6 and 12 months post-trial recruitment, receiving £5 gift vouchers for questionnaire completion. On completing the 1-month questionnaire, all participants would have been aware of their Early CDT^®^-Lung test result, but most positive result participants would have been unaware of their CT scan result.^16^ Test-positive participants also completed questionnaires at 18 and 24 months. Participants were withdrawn from the subsample on diagnosis of cancer, non-response to two consecutive follow-up questionnaires, on request or on trial withdrawal. Participants were included in the psychological outcomes analysis if they completed a 1- or 3-month questionnaire and until the point of withdrawal.

### Interventions

Blood samples were taken from all participants. The EarlyCDT^®^-Lung test was performed on intervention group samples. Participants with positive tests were invited to discuss their result with a research nurse and were told that the test detected 40/100 cases of lung cancer and that eight out of nine people testing positive do not have lung cancer. They received a CXR and low-dose CT scan followed by 6-monthly CT scans up to 2 years post-randomization. They were told the CT scan could reveal pulmonary nodules which are usually benign. CT scans were reviewed by a panel of experienced radiologists and respiratory physicians. Participants were followed up within the trial or in the National Health Service (NHS) care pathway as required.

Negative test participants were notified via letter, stating 98 to 99 out of 100 people with a negative test do not have lung cancer at the time of the test. They had no further trial investigations, received standard NHS care and were advised to seek medical attention for lung cancer symptoms.

### Outcomes

Data on age, gender, smoking history, lung cancer family history and Scottish Index of Multiple Deprivation was collected at trial recruitment. Data on education, marital status, employment and ethnic group was collected on the baseline questionnaire.

Affect was measured using the 20-item Positive and Negative Affect Schedule (PANAS).^17^ Emotional distress specific to lung cancer risk was assessed using the four-item Cancer Worry Scale (CWS) adapted for lung cancer.^18^ These were measured in all groups at baseline, 1, 3, 6 and 12 months and in the positive test group at 18 and 24 months. The Impact of Events scale (IES) assessed lung cancer screening distress,^19^ measured at 1, 3, 6 and 12 months in the positive and negative test groups only. Total IES score cut-off points for clinical levels of concern are: low < 8.5; medium: 8.6–19.0 and high > 19.^20^  Supplementary Table 1 provides details of the measures used.

### Sample size

Two hundred participants per group (positive test, negative test and control) would enable the detection of a difference in means in the IES between groups of 4.2 (standard deviation (SD): 15.04, which is based on unpublished data from the ProtecT prostate cancer study).^21^ To allow for attrition, we aimed to recruit 300 participants per group.

### Randomization

ECLS trial participants were randomized to intervention or CGs using the web-based Tayside Randomisation System (TRuST). Randomization was stratified by study site and was minimized by age, gender and smoking history.

### Blinding

Participants were not blinded to treatment group allocation.

### Statistical methods

Data analysis used Stata Statistical Software version 16.^22^ Continuous data were described using means and standard deviations (SDs) or medians and interquartile ranges (IQRs).Categorical variables were described using frequencies and percentages. As participants were a subsample of trial participants self-selecting to provide psychological outcomes’ data, baseline characteristics were compared between groups using Kruskal-Wallis tests for continuous data and Pearson’s chi-squared test for categorical data.

Multi-level regression compared outcomes between groups (positive, negative and control) at 1, 3, 6 and 12 months post-randomization by taking into account multiple observations per participant. Linear models were used for continuous variables and logistic models for categorical variables, adjusting for stratification and minimization variables and baseline values where measured. Model assumptions were checked by examining residuals plots. Outcome data were transformed to a logarithmic scale as necessary, with means (SDs) presented on original and logarithmic scales for ease of interpretation. Sensitivity analyses excluded observations with large residual values.

### Ethics approval

East of Scotland Research Ethics Committee (REC Number 13/ES/0024).

### Trial registration

ClinicalTrials.gov: NCT01925625.

## Results

Of 12 208 ECLS trial participants, 1079 were invited to provide psychological outcomes data. The final sample included in the analysis consisted of 1032 participants (95.6% follow-up rate; Supplementary Fig. 1). Groups were similar at baseline except that the negative test group had a higher proportion of males (54.6%) than the positive test (43.6%) and control (48.3%) groups (Table 1).

**Table 1:** Baseline characteristics of study participants

Characteristic (*N*, % unless stated otherwise)	Control (*n* = 350)	Test-positive (*n* = 321)	Test-negative (*n* = 361)	Comparison between groups
Study centre				*ꭓ* ^2^(2) = 1.23, *P* = 0.54
Glasgow	255 (72.9)	235 (73.2)	275 (76.2)	
Tayside	95 (27.1)	86 (26.8)	86 (23.8)	
Age				
50–54 years	76 (21.7)	62 (19.3)	88 (24.4)	*ꭓ* ^2^(8) = 6.11, *P* = 0.64
55–59 years	87 (24.9)	83 (25.9)	97 (26.9)	
60–64 years	80 (22.9)	62 (19.3)	72 (19.9)	
65–69 years	71 (20.3)	77 (24.0)	71 (19.7)	
70–75 years	36 (10.3)	37 (11.5)	33 (9.1)	
Gender				*ꭓ* ^2^(2) = 8.28, *P* = 0.02
Male	169 (48.3)	140 (43.6)	197 (54.6)	
Female	181 (51.7)	181 (56.4)	164 (45.4)	
Ethnic origin	[0]	[5]	[3]	*ꭓ* ^2^(2) = 0.30, *P* = 0.88
White British	344 (98.3)	309 (97.8)	350 (97.8)	
Other	6 (1.7)	7 (2.2)	8 (2.2)	
Marital status	[5]	[4]	[6]	*ꭓ* ^2^(6) = 5.36, *P* = 0.50
Single	43 (12.5)	25 (7.9)	38 (10.7)	
In a relationship	23 (6.7)	28 (8.8)	32 (9.0)	
Married/civil partnership	175 (50.7)	172 (54.3)	183 (51.5)	
Other	104 (30.1)	92 (29.0)	102 (28.7)	
Scottish Index of Multiple Deprivation	[4]	[0]	[0]	*ꭓ* ^2^(8) = 9.40, *P* = 0.31
1 (most deprived)	152 (43.9)	124 (38.6)	154 (42.7)	
2	60 (17.3)	82 (25.5)	64 (17.7)	
3	49 (14.2)	45 (14.0)	57 (15.8)	
4	48 (13.9)	41 (12.8)	50 (13.9)	
5 (least deprived	37 (10.7)	29 (9.0)	36 (10.0)	
Age on leaving full-time education (years)	[9]	[10]	[9]	*ꭓ* ^2^(2) = 0.43, *P* = 0.81
Median (IQR)	16.00 (1)	16.00 (1)	16.00 (1)	
Employment status	[7]	[6]	[6]	ꭓ^2^(6) = 5.78, *P* = 0.45
Paid employment	115 (33.5)	119 (37.8)	144 (40.6)	
Unable to work due to illness/disability	54 (15.7)	46 (14.6)	52 (14.6)	
Unemployed and looking for work	16 (4.7)	10 (3.2)	18 (5.1)	
Other	158 (46.1)	140 (44.4)	141 (39.7)	
Smoking status				*ꭓ* ^2^(2) = 4.49, *P* = 0.11
Smoker	189 (54.0)	165 (51.4)	214 (59.3)	
Ex-smoker	161 (46.0)	156 (48.6)	147 (40.7)	
Pack year history—median (IQR)	35.00 (21)	33.00 (23)	35.00 (22)	*ꭓ* ^2^(2) = 1.36, *P* = 0.51
First-degree relative with lung cancer				*ꭓ* ^2^(2) = 2.25, *P* = 0.33
Yes	87 (24.9)	95 (29.6)	92 (25.5)	
No	263 (75.1)	226 (70.4)	269 (74.5)	
PANAS positive	[31]	[33]	[40]	*ꭓ* ^2^(2) = 2.39, *P* = 0.30
Median (IQR)	34.00 (11)	35.00 (13)	34.00 (10)	
PANAS negative	[25]	[23]	[22]	*ꭓ* ^2^(2) = 0.66, *P* = 0.72
Median (IQR)	13.00 (7)	13.00 (6)	14.00 (7)	
LCWS—frequency of worry	[1]	[4]	[1]	*ꭓ* ^2^(2) = 2.63, *P* = 0.27
Not worried	131 (37.5)	138 (43.5)	150 (41.7)	
Worried	218 (62.5)	179 (55.8)	210 (58.3)	
LCWS—impact of worry	[4]	[4]	[3]	*ꭓ* ^2^(2) = 2.76, *P* = 0.25
Median (IQR)	2.00 (1)	2.00 (1)	3.00 (1)	
LCWS—worry about tests	[3]	[4]	[0]	*ꭓ* ^2^(2) = 1.95, *P* = 0.38
Not anxious	314 (90.5)	291 (91.8)	320 (88.6)	
Anxious	33 (9.5)	26 (8.2)	41 (11.4)	

[] missing values. IQR = interquartile range.

Boxplots for psychological outcomes are shown in Supplementary Fig. 2. Comparisons of psychological outcomes between positive and negative test groups, up to 12 months, are shown in Table 2. Unless stated otherwise, effect sizes for scores are differences between means. Total IES scores are described below, but not subscale scores, as findings were similar to total IES scores.

**Table 2:** Psychological outcomes in the positive test (+ve) and negative test (−ve) groups from 1 to 12 months

	1 m			3 m			6 m			12 m			
	+ve test, *M* (SD)	−ve test, *M* (SD)	Adj. diff. in means [95% CI]	+ve test, *M* (SD)	−ve test, *M* (SD)	Adj. diff. in means [95% CI]	*+*ve test, *M* (SD)	−ve test, *M* (SD)	Adj. diff. in means [95% CI]	+ve test, *M* (SD)	−ve test, *M* (SD)	Adj. diff. in means [95% CI]	*P-*value for diff. between groups over time
PANAS positive	31.87 (8.62)	31.82 (8.19)	−0.82 [−1.97, 0.32]	30.29 (9.14)	30.91 (8.29)	−1.49 [−2.65, −0.33]	30.90 (9.68)	31.10 (8.40)	−0.84 [−2.00, 0.32]	31.11 (9.92)	30.90 (8.27)	−0.43 [−1.59, 0.73]	0.32
PANAS negative	16.47 (7.15)	16.36 (7.45)		16.17 (7.25)	15.94 (6.94)		16.02 (6.97)	16.55 (7.33)		16.23 (7.61)	16.54 (7.01)		
PANAS negative (log)	2.72 (0.38)	2.71 (0.38)	0.03 [−0.03, 0.09]	2.70 (0.39)	2.69 (0.37)	0.02 [−0.04, 0.07]	2.70 (0.37)	2.73 (0.38)	−0.01 [−0.08, 0.04]	2.70 (0.39)	2.73 (0.38)	−0.00 [−0.06, 0.06]	0.22
IES—total Score	23.16 (9.50)	19.68 (7.85)	3.59 [2.28, 4.90]	21.51 (8.90)	19.57 (7.87)	2.29 [0.97, 3.61]	20.90 (9.02)	19.55 (7.76)	1.94 [0.61, 3.27]	19.73 (7.74)	19.64 (8.04)	0.99 [−0.35, 2.33]	<0.001
IES—intrusion	10.51 (4.38)	9.03 (3.64)	1.54 [0.95, 2.14]	9.87 (4.10)	8.97 (3.64)	1.07 [0.48, 1.67]	9.54 (4.12)	8.98 (3.65)	0.76 [0.16, 1.36]	8.95 (3.48)	9.09 (3.73)	0.10 [−0.50, 0.71]	<0.001
IES—avoidance	12.76 (5.59)	10.82 (4.60)	2.04 [1.27, 2.80]	11.66 (5.18)	10.66 (4.45)	1.18 [0.41, 1.95]	11.50 (5.38)	10.67 (4.34)	1.17 [0.40, 1.94]	10.96 (4.72)	10.59 (4.61)	0.74 [−0.04, 1.52]	0.003
LCWS—impact	3.39 (1.62)	3.27 (1.62)	0.26 [0.05, 0.47]	3.34 (1.60)	3.17 (1.62)	0.28 [0.07, 0.50]	3.29 (1.60)	3.30 (1.69)	0.16 [−0.06, 0.37]	3.22 (1.66)	3.32 (1.77)	0.07 [−0.15, 0.29]	0.14
	+ve test, *N* (%)	−ve test, *N* (%)	OR [95% CI]	+ve test, *N* (%)	−ve test, *N* (%)	OR [95% CI]	+ve test, *N* (%)	−ve test, *N* (%)	OR [95% CI]	+ve test, *N* (%)	−ve test, *N* (%)	OR [95% CI]	
LCWS—frequency	200 (64.52)	201 (57.59)	2.52[1.31, 4.83]	186 (61.59)	185 (55.72)	2.43 [1.26, 4.68]	191 (64.09)	181 (56.39)	2.87 [1.48, 5.60]	168 (58.74)	178 (56.87)	1.68 [0.86, 3.27]	0.50
LCWS—worry	73 (23.55)	47 (13.51)	5.79 [2.66, 12.63]	55 (18.27)	53 (16.01)	2.08 [0.95, 4.56]	54 (18.24)	60 (18.69)	1.45 [0.67, 3.14]	50 (17.48)	63 (20.32)	1.08 [0.50, 2.35]	<0.001

Adjusted for study site (Greater Glasgow and Clyde, Tayside), minimization variables (age group, gender and smoking history) and baseline values where measured. *M* = mean. 1m = 1 month, 3m = 3 months, 6m = 6 months, 12m = 12 months.

### Comparing positive and negative test groups

The positive test group had lower positive affect scores at 3 months (−1.49, 95% confidence interval (CI): −2.65, −0.33), reported greater impact of worries on daily functioning at 1 month (0.26, 95% CI: 0.05, 0.47) and 3 months (0.28, 95% CI: 0.07, 0.50) and greater lung cancer screening distress at 1 month (3.59, 95% CI: 2.28, 4.90), 3 months (2.29, 95% CI: 0.97, 3.61) and 6 months (1.94, 95% CI: 0.61, 3.27) compared to the negative test group. The positive test groups were also more likely to frequently worry about lung cancer at 1 month (odds ratio (OR): 2.52, 95% CI: 1.31, 4.83), 3 months (OR: 2.43, 95% CI: 1.26, 4.68) and 6 months (OR: 2.87, 95% CI: 1.48, 5.60) and have greater worry about tests at 1 month (OR: 5.79, 95% CI: 2.66, 12.63). Outcomes varied over time between groups for lung cancer screening distress (*P* < 0.001), reducing over time in the positive test group but remaining stable in the negative test group. Worry about tests reduced over time in the positive test group and increased over time in the negative test group (*P* < 0.001). The percentage of participants with IES total scores >19 in the positive test group reduced from 49 to 28% between 1 and 12 months and remained stable in the negative test group (30 and 29% respectively).

### Comparing positive test and CGs

Comparisons of psychological outcomes between positive test and CGs are shown in Table 3. The only difference between the groups was that the positive test group were more likely to worry about tests at 1 month (OR: 3.40, 95% CI: 1.69, 6.84). Worry about tests reduced over time in the positive test group and increased over time in the CG (*P* = 0.002).

**Table 3:** Psychological outcomes in the positive test (+ve) and CGs (no test) from 1 to 12 months

	1m			3m			6m			12m			
	+ve test, *M* (SD)	No test, *M* (SD)	Adj. diff. in means [95% CI]	+ve test, *M* (SD)	No test, *M* (SD)	Adj. diff. in means [95% CI]	+ve test, *M* (SD)	No test, *M* (SD)	Adj. diff. in means [95% CI]	+ve test, *M* (SD)	No test, *M* (SD)	Adj. diff. in means [95% CI]	*P*-value diff. between groups over time
PANAS positive	31.87 (8.62)	30.61 (8.50)	0.54 [−0.60, 1.57]	30.29 (9.14)	30.00 (8.39)	−0.20 [−1.34, 0.94]	30.90 (9.68)	30.15 (9.25)	0.28 [−0.85, 1.43]	31.11 (9.92)	30.43 (9.49)	0.45 [−0.71, 1.60]	0.56
PANAS negative	16.47 (7.15)	16.94 (7.53)		16.17 (7.25)	17.29 (7.83)		16.02 (6.97)	17.03 (8.00)		16.23 (7.61)	16.97 (8.10)		
PANAS negative (log)	2.72 (0.38)	2.75 (0.39)	−0.01 [−0.07, 0.04]	2.70 (0.39)	2.76 (0.40)	−0.06 [−0.12, 0.00]	2.70 (0.37)	2.75 (0.40)	−0.03 [−0.09, 0.03]	2.70 (0.39)	2.74 (0.41)	−0.03 [−0.09, 0.03]	0.29
LCWS—impact	3.39 (1.62)	3.28 (1.63)	0.13 [−0.08, 0.34]	3.34 (1.60)	3.31 (1.60)	0.01 [−0.20, 0.22]	3.29 (1.60)	3.30 (1.64)	0.01 [−0.22, 0.21]	3.22 (1.66)	3.35 (1.60)	−0.12 [−0.33, 0.09]	0.09
	+ve test, *N* (%)	No test, *N* (%)	OR [95% CI]	+ve test, *N* (%)	No test, *N* (%)	OR [95% CI]	+ve test, *N* (%)	No test, *N* (%)	OR [95% CI]	+ve test, *N* (%)	No test, *N* (%)	OR [95% CI]	
LCWS—frequency	200 (64.52)	214 (62.76)	1.65 [0.84, 3.21]	186 (61.59)	207 (62.35)	1.17 [0.60, 2.29]	191 (64.09)	204 (62.01)	1.61 [0.82, 3.16]	168 (58.74)	196 (61.64)	0.99 [0.50, 1.95]	0.45
LCWS—worry	73 (23.55)	52 (15.25)	3.40 [1.69, 6.84]	55 (18.27)	56 (16.92)	1.43 [0.70, 2.91]	54 (18.24)	57 (17.48)	1.30 [0.64, 2.65]	50 (17.48)	68 (21.52)	0.71 [0.35, 1.44]	0.002

Adjusted for study site (Greater Glasgow and Clyde, Tayside), minimization variables (age group, gender and smoking history) and baseline values where measured. *M* = mean. 1m = 1 month, 3m = 3 months, 6m = 6 months, 12m = 12 months.

### Comparing negative test and CGs

Comparisons of psychological outcomes between the negative test and CGs are shown in Table 4. The negative test group had lower negative affect scores at 3 months (log-transformed: −0.08, 95% CI: −0.13, −0.02), higher positive affect scores at 1 month (1.52, 95% CI: 0.43, 2.61), 3 months (1.43, 95% CI: 0.33, 2.53) and 6 months (1.27, 95% CI: 0.17, 2.37). The negative test group also reported less impact of worries on daily functioning at 3 months (−0.27, 95% CI: −0.48, −0.07) and were less likely to frequently worry about lung cancer at 3 months (OR: 0.49, 95% CI: 0.26, 0.92). None of the outcome measures varied significantly over time between the negative test and CGs.

**Table 4:** Psychological outcomes in the negative test (−ve) and CGs (no test) from 1 to 12 months

	1m			3m			6m			12m			
	−ve test, *M* (SD)	No test, *M* (SD)	Adj. diff. in means [95% CI]	−ve test, *M* (SD)	No test, *M* (SD)	Adj. diff. in means [95% CI]	−ve test, *M* (SD)	No test, *M* (SD)	Adj. diff. in means [95% CI]	−ve test, *M* (SD)	No test, *M* (SD)	Adj. diff. in means [95% CI]	*P*-value diff. between groups over time
PANAS positive	31.82 (8.19)	30.61 (8.50)	1.52 [0.43, 2.61]	30.91 (8.29)	30.00 (8.39)	1.43 [0.33, 2.53]	31.10 (8.40)	30.15 (9.25)	1.27 [0.17, 2.37]	30.90 (8.27)	30.43 (9.49)	1.03 [−0.09, 2.14]	0.80
PANAS negative	16.36 (7.45)	16.94 (7.53)		15.94 (6.94)	17.29 (7.83)		16.55 (7.33)	17.03 (8.00)		16.54 (7.01)	16.97 (8.10)		
PANAS negative (log)	2.71 (0.38)	2.75 (0.39)	−0.05 [−0.11, 0.01]	2.69 (0.37)	2.76 (0.40)	−0.08 [−0.13, −0.02]	2.73 (0.38)	2.75 (0.40)	−0.02 [−0.08, 0.04]	2.73 (0.38)	2.74 (0.41)	−0.03 [−0.09, 0.03]	0.09
LCWS—impact	3.27 (1.62)	3.28 (1.63)	−0.13 [−0.33, 0.07]	3.17 (1.62)	3.31 (1.60)	−0.27 [−0.48, −0.07]	3.30 (1.69)	3.30 (1.64)	−0.17 [−0.37, 0.04]	3.32 (1.77)	3.35 (1.60)	−0.19 [−0.40, 0.01]	0.48
	−ve test, *N* (%)	No test, *N* (%)	OR [95% CI]	−ve test, *N* (%)	No test, *N* (%)	OR [95% CI]	−ve test, *N* (%)	No test, *N* (%)	OR [95% CI]	−ve test, N (%)	No test, *N* (%)	OR [95% CI]	
LCWS—frequency	201 (57.59)	214 (62.76)	0.66 [0.35, 1.23]	185 (55.72)	207 (62.35)	0.49 [0.26, 0.92]	181 (56.39)	204 (62.01)	0.56 [0.29, 1.07]	178 (56.87)	196 (61.64)	0.59 [0.31, 1.14]	0.86
LCWS—worry	47 (13.51)	52 (15.25)	0.63 [0.28, 1.39]	53 (16.01)	56 (16.92)	0.69 [0.32, 1.51]	60 (18.69)	57 (17.48)	0.92 [0.43, 1.98]	63 (20.32)	68 (21.52)	0.65 [0.31, 1.37]	0.80

Adjusted for study site (Greater Glasgow and Clyde, Tayside), minimization variables (age group, gender and smoking history) and baseline values where measured. *M* = mean. 1m = 1 month, 3m = 3 months, 6m = 6 months, 12m = 12 months

Psychological outcome measures at 18 and 24 months in the test-positive group were similar to those at 12 months (Supplementary Table 2). A small number of findings (4 out of 90) were not robust to excluding observations with large residual values (Supplementary Table 3).

## Discussion

### Main findings

This is the first trial to report psychological outcomes following a novel tumour antibody blood test for lung cancer prior to CT scanning for those with a positive test. We found negative psychological effects in those with a positive test and positive effects in those with a negative test up to 6 months post-screening. However, differences in most outcomes were small and were unlikely to be clinically important. Overall, there was no long-term psychological impact for the majority of those screened, with the exception of lung cancer screening distress for which potentially clinically concerning scores were reported by 28% of the test-positive and 29% of the test-negative groups 12 months post-screening.

### What is already known on this topic

Similar to our findings, trials report greater psychological burden (e.g. higher state anxiety and distress) up to 6 months post-screening in those with positive compared to those with negative screening results,^6–9^ with distress reducing to similar levels as the test-negative group by 12 months.^6^^,^^7^ However, our finding of potentially concerning levels of lung cancer screening distress in almost one third of those screened at 12 months differs from the NELSON trial that found much lower levels of distress using the IES.^10^^,^^11^ Our trial population was similar to the NELSON trial, and although our trial included more investigations (blood test, then CXR and serial CT scans for those with positive tests), this is unlikely to explain the differences in distress levels, as those with a negative test had only one investigation. One possible explanation is that the NELSON trial tailored the IES questions to lung cancer distress while we tailored ours to lung cancer ‘screening’ distress.

Unlike previous trials, we included a positive psychological outcome measure, showing greater short-term positive affect in the negative test group than either comparison group. We also found a short-term reduction in negative affect in the negative test group compared to the CG, which is similar to the NELSON trial finding of short-term decreases in distress in those with a negative screening result.^10^ Reassurance provided by a negative test may possibly explain these findings.

The DLCST and UKLS found less favourable psychological outcomes for the CG. DLCST CG participants experienced higher anxiety, self-blame and dejection than the CT screening group.^13^ UKLS trial CG participants reported higher levels of anxiety and depression than the screened group up to 2 years post-CT scan; however, similar to our trial, differences were small and clinically unimportant.^9^ It is possible that participants in these trials have a heightened awareness of lung cancer risk, and CG participants may be disappointed by not gaining reassurance from screening. By contrast, the NELSON trial found no difference between screened and CGs in quality of life, anxiety and lung cancer-specific distress 1.5 years following screening.^11^

### What this study adds

Most individuals will not experience negative psychological outcomes after a novel antibody blood test followed by imaging, although some with a positive blood test may do so up to 6 months post-screening. Our study was the first to measure lung cancer screening distress, and we found that almost one third of those screened, regardless of their blood test result, may experience lung cancer screening distress up to 1 year post-screening. If lung cancer screening using the Early CDT^®^-Lung test is implemented in the UK, consideration should be given to identifying psychological distress related to screening and providing psychological support if required.

### Limitations of this study

We used several psychological measures resulting in numerous statistical tests with potential for Type 1 error; hence, findings must be interpreted with this in mind. Lung cancer screening distress could only be measured in those screened (i.e. received the blood test); hence, we do not have scores prior to screening or for a CG for comparison. In our trial, the test-negative and CGs only completed measures up to 12 months post-screening, so longer-term impacts are unknown. Further research is required to confirm our findings regarding lung cancer screening distress, both in trials using biomarkers and in trials of CT screening without the use of biomarkers. Further research is also required to measure longer-term positive and negative psychological outcomes in screened and unscreened participants.

In conclusion, screening for lung cancer using a novel tumour antibody blood test followed by a CT scan for those testing positive does not appear to have long-term adverse psychological impacts for most of those screened. This approach may have advantages over CT screening in terms of radiation exposure, costs and ease of administration and may therefore be particularly useful in settings where health care services are more poorly resourced and/or access to CT scanning is limited.

## Authors’ contributions

DK, KV, JFRR, RdN, FMS, FSM, SS and RL conceived and designed the study. LB and BY collected data. DK, KV, JFRR, RdN, LB and BY conceived and designed the analysis. JH, KA and DK analysed and interpreted data. JH, KA and DK drafted the manuscript. All authors critically revised the manuscript and approved the version to be published.

## Funding

This work was supported by the Scottish Government and Oncimmune Ltd. Follow-up data collection for psychological outcome measures was supported by Oncimmune Ltd.

## Competing interests

FMS, FSM, RL report grants from Oncimmune Ltd. and grants from Scottish Government Health and Social Care Directorate of the Chief Scientist Office (CSO), during the conduct of the study.

SS reports grants from Oncimmune Ltd. and grants from Scottish Government Health and Social Care Directorate of the Chief Scientist Office (CSO), during the conduct of the study and non-financial support from Glaxosmithkline, non-financial support from Astra Zeneca, outside the submitted work.

JFRR was a founder of Oncimmune, a company spun out from the University of Nottingham based on his academic research. Professor Robertson is a named inventor on 7 families of patents - some have been issued and other pending. Between 2003–2013 he was Chief Scientific Officer of Oncimmune and a Director of the company. During this time he was responsible for the original drafting of the ECLS protocol. Since 2013 he have had no involvement in the science or management of the company. He has been and remains a shareholder in the company.

RdN, LB, BY, JH, KA report no competing interests.

DK and KV report a grant from Oncimmune Ltd. for collection of follow-up data on psychological and behavioural outcomes during the conduct of the study.

## Supplementary Material

Supplementary_tables_2Feb22_CLEAN_fdac032

CONSORT-2010-Checklist-MS-Word_(1)_fdac032

## Notes

**Hancox J.,** Assistant Professor

**Ayling K.,** Senior Research Fellow and Assistant Professor

**Bedford L.,** Honorary Research Associate

**Vedhara K.,** Professor of Health Psychology

**Roberston J. F. R.,** Professor of Surgery

**Young B.,** PhD Student

**das Nair R.,** Professor of Clinical Psychology and Neuropsychology

**Sullivan F. M.,** Professor of Primary Care Medicine

**Schembri S.,** Consultant Physician in Respiratory and General Internal Medicine Respiratory Medicine

**Mair F. S.,** Professor of Primary Care Research

**Littleford R.,** Clinical Trials Research Specialist

**Kendrick D.,** Professor of Primary Care Research
