# Supplementary material for: Psychological impact of lung cancer screening using a novel antibody blood test followed by imaging: the ECLS randomized controlled trial
Source: J Public Health (Oxf). 2022 Mar 14;45(2):e275–84. doi: 10.1093/pubmed/fdac032 (PMC10273385; doi:10.1093/pubmed/fdac032)
Supplement: Supplementary_tables_2Feb22_CLEAN_fdac032 [file supplementary_tables_2feb22_clean_fdac032.docx]

**Supplementary table 1: Psychological outcome measures used in the study.**

| Measure | Description | Cronbach’s alpha |
| --- | --- | --- |
| Positive and negative affect scale | PANAS has two subscales: 10 items measuring positive affect (e.g., excited, enthusiastic, inspired) and 10 measuring negative affect (e.g., distressed, upset, jittery). Participants rated the extent to which they felt each emotion over the past week using a 5-point Likert scale (1 = very slightly/not at all, 5 = extremely). Subscale scores were summed to create scores for positive and negative affect. | Positive affect α=0.92  Negative affect α=0.91 |
| 4-item Cancer Worry Scale (CWS) adapted for lung cancer | Items measured (1) frequency of worries, (2) impact of worries on mood, (3) impact of worries on daily functioning and (4) anxiety related to future testing using 5-point Likert scales (1 = not at all; 5 = almost all the time). Frequency of worry (item 1) was dichotomised into not worried (not at all worried/rarely worried) vs. worried (sometimes worried/often worried/worried almost all of the time).  Scores from items 2 and 3 were summed to create a continuous impact of worry variable.  Worry about tests (item 4) was dichotomised into not anxious (not at all anxious/a little anxious) vs. anxious (somewhat anxious/anxious a lot/anxious almost all of the time). | Impact of worry α=0.82 |
| The 15-Item Impact of Events Scale | The IES has two subscales: intrusion (7 items, e.g. “I thought about being tested for lung cancer when I did not mean to‟) and avoidance (8 items, e.g., “I tried to remove being tested for lung cancer from memory‟). Participants reported frequency of each feeling during the past week on a 4-point Likert scale (0 = not at all, 1 = rarely, 3 = sometimes, 5 = often). Responses were summed to create subscale and total scores (maximum = 75). Total IES score cut-off points for clinical levels of concern are: low< 8.5; medium 8.6-19.0; and high >19.[1] | 15-Item IES α=0.94 |

[1] Joseph S. Psychometric evaluation of Horowitz's Impact of Event Scale: a review. *J Trauma Stress.* 2000;13(1):101-13

**Supplementary table 2:** Psychological outcomes in the positive test group from 1 to 24 months

|  | ***1 Month***  ***M (SD)*** | ***3 Months***  ***M (SD)*** | ***6 Months***  ***M (SD)*** | ***12 Months***  ***M (SD)*** | ***18 Months***  ***M (SD)*** | ***24 Months***  ***M (SD)*** |
| --- | --- | --- | --- | --- | --- | --- |
| **PANAS Positive** | 31.87  (8.62) | 30.29  (9.14) | 30.90  (9.68) | 31.11  (9.92) | 31.47  (9.43) | 31.19  (9.11) |
| **PANAS Negative** | 16.47  (7.15) | 16.17  (7.25) | 16.02  (6.97) | 16.23  (7.61) | 16.72  (7.64) | 16.16  (7.60) |
| **LCWS-Impact** | 3.39  (1.62) | 3.34  (1.60) | 3.29  (1.60) | 3.22  (1.66) | 3.10  (1.53) | 3.19  (1.63) |
|  | ***1 Month***  ***M (SD)*** | ***3 Months***  ***M (SD)*** | ***6 Months***  ***M (SD)*** | ***12 Months***  ***M (SD)*** | ***18 months***  ***N (%)*** | ***24 months***  ***N (%)*** |
| **LCWS-Frequency** | 200  (64.52) | 186  (61.59) | 191  (64.09) | 168  (58.74) | 152  (56.72) | 141  (55.29) |
| **LCWS-Worry** | 73  (23.55) | 55  (18.27) | 54  (18.24) | 50  (17.48) | 45  (16.92) | 48  (18.75) |

M=mean, SD=standard deviation

**Supplementary table 3**. Sensitivity analyses where findings were not robust to excluding cases with large residual values.

|  | Main analysis | Sensitivity analysis |
| --- | --- | --- |
| ***Positive test group versus negative test group*** | | |
| PANAS Positive at 3 months | -1.49, 95%CI -2.65, -0.33 | -0.79, 95%CI -1.83, 0.26 |
| IES total score at 12 months | 0.99, 95%CI -0.35, 2.33 | 1.33, 95%CI 0.04, 2.61 |
| ***Negative test group versus control group*** | | |
| PANAS Positive at 12 months | 1.03, 95%CI -0.09, 2.14 | 1.22, 95%CI 0.21, 2.22 |
| LCWS-Impact at 12 months | -0.19, 95%CI -0.40, 0.01 | -0.21, 95%CI -0.41, -0.01 |

Adjusted for stratification (Greater Glasgow and Clyde, Tayside and Lanarkshire) and minimisation variables (age group, gender, smoking history) and baseline values where measured
